# Long-tunneled external ventricular drainage (LTEVD) for the prevention and treatment of infections in pediatric and adult hydrocephalus

**DOI:** 10.1007/s00701-025-06677-3

**Published:** 2025-10-10

**Authors:** Mateo Tomas Fariña Nuñez, Veronica Percuoco, Lara Maria Höbner, Stefanos Voglis, Richard Parvin, Massimo Barbagallo, Adrian Elmi Terander, Erik Edström, Carlo Serra, Luca Regli, Victor E. Staartjes, Flavio Vasella

**Affiliations:** 1 Machine Intelligence in Clinical Neuroscience & Microsurgical Neuroanatomy (MICN) Laboratory, Department of Neurosurgery, Clinical Neuroscience Center, University Hospital Zurich, University of Zurich, Zurich, Switzerland; 2 Department of Orthopaedic Surgery, Neurosurgery and Spine Surgery, Schulthess Clinic, Zurich, Switzerland; 3 Department of Neurosurgery, Klinik St. Anna Hirslanden, Lucerne, Switzerland; 4 Department of Neurology, Klinik Hirslanden Zürich, Zurich, Switzerland; 5 Department of Clinical Neuroscience, Karolinska Institutet, Stockholm, Sweden; 6 Capio Stockholm Spine Center, Löwenströmska Hospital, Upplands Väsby, Sweden

**Keywords:** Infection, Cerebrospinal fluid infection, Long-tunneled external ventricular drainage, Short-tunneled external ventricular drainage, Subcutaneous tunnel drainage, Ventriculostomy

## Abstract

**Background:**

Long-tunneled external ventricular drainage (LTEVD) has been proposed as an alternative to conventional short-tunneled external ventricular drainage (STEVD), particularly to reduce complications such as catheter dislocation and secondary infections. Despite potential advantages, awareness of LTEVD and high-quality literature remain limited. This systematic review and meta-analysis intends to evaluate the safety and efficacy of LTEVD in pediatric and adult populations.

**Methods:**

A systematic review was conducted to identify studies including both adult and pediatric patients undergoing LTEVD and reporting intra-, peri-, and postoperative clinical outcomes, indications, and complication rates. Major scientific databases were searched for articles published up to 2024. Two authors independently extracted data.

**Results:**

Fifteen studies (n = 1,024; 612 adults, 412 children) met inclusion criteria, with most applying LTEVD to manage infections. The mean tunneling length was 41.3 cm, and the median duration of drainage was 7 days. LTEVD removal was performed in the operating room in 87% of cases, compared to 92% bedside removals for STEVD. Six studies (n = 123) reported conversion to ventriculoperitoneal shunt. Meta-analysis of infection rates (10 studies, n = 824) showed no statistically significant difference between LTEVD and STEVD (OR 1.83, 95%CI 0.84–3.99; p = 0.13). Blockage and dislocation rates were comparable (LTEVD 2.7% vs. STEVD 7.0%, p = 0.075; LTEVD 5.4% vs. STEVD 2.3%, p = 0.151).

**Conclusion:**

Outcomes for LTEVD are generally comparable to STEVD in both children and adults. While LTEVD may offer potential long-term advantages for infection management, current evidence does not confirm its superiority. LTEVD may be appropriate for prolonged drainage needs, but further research is required due to limited evidence and study heterogeneity.

## Introduction

External ventricular drainage (EVD) insertion is among the most performed neurosurgical procedures worldwide, essential for managing a variety of acute neurosurgical conditions including hydrocephalus, intraventricular hemorrhage, intracranial hypertension, and central nervous system (CNS) infections that necessitate immediate cerebrospinal fluid (CSF) diversion [6, 14]. In its conventional form, neurosurgeons insert a ventricular catheter through a burr hole into the lateral ventricle, tunneling the distal catheter subcutaneously a relatively short distance from the entry point, commonly referred to as short-tunneled external ventricular drainage (STEVD) [14, 25]. Although STEVD is considered a relatively straightforward and standardized procedure, complications remain frequent and challenging. Among these complications, catheter-related infections, catheter blockage, misplacement, inadvertent dislocation, and CSF leakage are particularly problematic [3, 17, 19].

Catheter-related infections represent a major source of morbidity, leading to prolonged hospital stays, additional surgical interventions, higher healthcare costs, and significantly worse patient outcomes [19]. The incidence of catheter-related infections following STEVD placement varies considerably across different Healthcare systems and has been reported to be as high as 32% in developing regions [21]. These infections typically arise from microbial colonization and migration along the external catheter segment, progressing into the ventricles and CSF compartment. Despite substantial efforts, including the introduction of antibiotic-coated catheters, rigorous infection control measures, shortening the duration of drainage, or prophylactic catheter exchange every five to seven days, significant infection rates persist. Furthermore, the frequent bedside handling associated with STEVD management further increases infection risk, complicating clinical management and negatively affecting patient recovery [3, 17, 19].

In 1995, Khanna and colleagues introduced a novel concept termed long-tunneled external ventricular drainage (LTEVD), designed specifically to reduce EVD-related complications, primarily infections [14]. These advantages may be particularly relevant in patients with e.g. shunt infections who require long-term external drainage. Unlike STEVD, LTEVD involves creating a significantly longer subcutaneous catheter tunnel, typically 40 to 60 cm, to increase the physical separation between the catheter’s entry into the ventricular system and the external exit point, most frequently placed on the abdomen or thorax. This increased distance was hypothesized to substantially decrease bacterial colonization and migration risks, consequently reducing infection rates [6, 14, 16, 19, 25, 29]. Despite encouraging early outcomes, LTEVD did not gain widespread acceptance and remains relatively underutilized in neurosurgical practice, likely due to its perceived technical complexity, longer operative times, and the frequent requirement for surgical removal under general anesthesia due to implanted burr hole reservoirs [9, 16, 29].

Given the clinical significance of EVD-related complications and considering the potential, yet uncertain advantages offered by LTEVD, a thorough and objective analysis of current evidence is warranted. To date, no systematic review or meta-analysis has specifically evaluated the comparative outcomes of LTEVD versus STEVD across pediatric and adult populations. Clarifying this uncertainty could potentially inform clinical practice, as even modest improvements in complication rates might translate into clinically meaningful patient benefits and improved healthcare resource utilization.

Therefore, the primary aim of this systematic review and meta-analysis was to critically evaluate the available evidence regarding LTEVD compared with STEVD, specifically examining safety profiles and efficacy in reducing postoperative infections, catheter blockage, dislocation, and CSF leakage. Additionally, this analysis aimed to identify existing knowledge gaps and to propose directions for future research, potentially facilitating evidence-based clinical decision-making and procedural standardization.

## Materials and methods

### Overview and data collection

A systematic review of the literature was conducted to identify any studies presenting both adult and pediatric patient cohorts undergoing LTEVD and reporting intra-/perioperative clinical outcomes, including (1) infection, (2) blockage, (3) dislocation, (4) other complications (including e.g. CSF leakage), (5) revision surgeries/operations including EVD removal, (6) duration of EVD drainage, (7) long-term CSF diversion techniques and (7) mortality.

Title and abstract screening, full-text review, and data extraction were handled independently by two reviewers (MTFN and VP) using Covidence (Covidence systematic review software, Melbourne, Australia), and disagreements at any stage were resolved by discussion and consensus, or if not resolved by discussion with a third reviewer (VES). We used the Arksey and O’ Malley framework [7] and PRISMA [22].

### Search strategy

A systematic search was performed to identify eligible articles using the electronic databases PubMed/MEDLINE, Embase, and Scopus from inception to January 29, 2024. A comprehensive search strategy was designed using relevant medical subject heading (MeSH) terms and key words as follows: (“external ventricular drain” OR “external ventricular drainage” OR “ventriculostomy” OR “EVD”) AND (“long-tunneled” OR “long-tunneling” OR “extended subcutaneous” OR “tunnel” OR “tunneling” OR “tunnelling”). Word variations and exploded medical subject Headings were searched for whenever feasible. References were screened to identify additional relevant articles. Additional studies were identified by manually screening reference lists of relevant articles and reviews. The search included studies published up to January 2024 with no backwards limitations, to capture the historical evolution and contemporary clinical outcomes associated with LTEVD.

### Study selection

Only in vivo studies were considered, involving either adult or pediatric populations. Prospective and retrospective studies, randomized control trials (RCT), and case reports were included. Both comparative and non-comparative studies were considered. Systematic reviews and studies reporting data exclusively on short tunneled EVD (STEVD) were excluded. To be considered, patients had to undergo LTEVD alone (primary tunneling) or secondary to STEVD (secondary tunneling). To be eligible for inclusion, studies had to assess at least one of the above-mentioned variables of interest as preoperative, intraoperative, and postoperative time points.

### Data extraction and quality assessment

The following data were extracted from all included publications: Study design and year of publication, number of patients, mean patient age and sex distribution, including, indication for EVD insertion, underlying pathology, criteria for LTEVD versus STEVD insertion, primary or secondary LTEVD insertion, length of tunneling, externalization site, reservoir, valve, as well as at least one of the outcomes of interest. The methodological quality of the included studies was graded using the Newcastle–Ottawa Quality Assessment Scale for Cohort Studies [28] and the Cochrane Risk-of-Bias (RoB-2) tool for RCTs [11].

### Statistical meta-analysis

Statistical analyses were conducted using R [23]. Based on the anticipated heterogeneity and the lack of larger RCTs for certain outcomes, a random-effects analysis model was applied (Mantel–Haenszel). Cochran’s Q and I^2^ were used to evaluate the strength of evidence for heterogeneity and the level of heterogeneity, respectively. A p ≤ 0.1 was considered as relevant heterogeneity. Publication bias was assessed by funnel plots. P ≤ 0.05 was considered statistically significant for the assessment of overall effect. For key outcomes where insufficient data were available to support a model-based meta-analysis, specifically dislocation and blockage rates, pooled proportions were calculated to enable group comparisons. Intergroup differences between STEVD and LTEVD were assessed using Chi-square tests applied to the aggregated data.

## Results

### Literature search

**Fig. 1:**
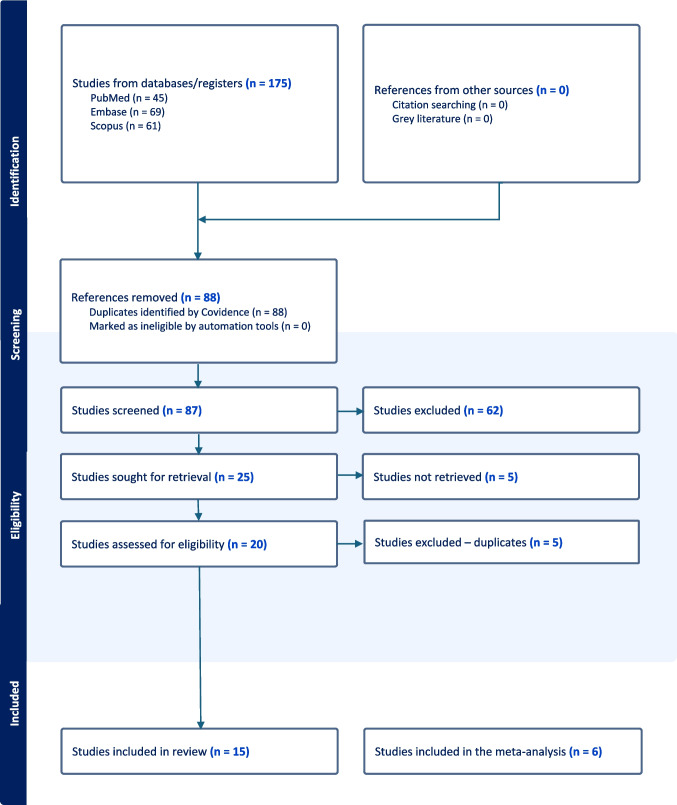
PRISMA Flowchart

We identified a total of 15 eligible studies for qualitative analysis [4, 6, 8–10, 13, 14, 16, 18, 20, 25–27, 29, 30]. Six comparative studies (n = 824) were eligible for inclusion in the quantitative meta-analysis, as they specifically compared LTEVD to STEVD and provided sufficient data for statistical evaluation [4, 9, 18, 25, 27, 29]. The detailed PRISMA flowchart illustrating study selection and inclusion is presented in Fig. 1. Only for infection rates we assembled enough data from comparative studies to enable a meta-analysis.

### Included study characteristics and quality assessment

A detailed summary of included studies, including study design, patient demographics and outcomes assessed, is presented in Table 1. Clinical outcomes extracted from the included studies are detailed in Table 2. Further study-level details, methodological considerations and quality assessment results, are provided in Table 3. The following studies were identified for qualitative assessment:

- Studies on LTEVD alone as either primary or secondary intervention. 5 were case reports [8, 10, 13, 20, 30] and 3 retrospective studies [6, 14, 16]. High risk of bias exists in this cohort due to poor reproducibility.
- Studies comparing LTEVD to a STEVD population. Also, in this group a high risk of bias exists. 2 studies were prospective and non-randomized control trials (non RCT) [18, 29], 1 quasi experimental [25] and the remaining were retrospective studies [4, 9, 26, 27].

**Table 1:** Overview of the basic study data of the 15 included studies

**Authors**	P**atients N**	**Population**	**Indication**	**Pathology**	**Secondary LTEVD**	**Follow-up (months)**	**Tunneling cm**	**Externalization**	**Reservoir**	**Valve**
LTEVD										
Waqas et al	2 Patients	Pediatric	OH	Cerebritis with brain edema	0	18 ± 6	N/A	N/A	N/A	N/A
Nakano et al	1 Patients	Pediatric	OH	IVH	1 (100%) After VPS	1	N/A	Abdomen	N/A	N/A
Collins et al	177 Patients 181 LTEVD	Pediatric	129 (77%): OH 48 (27%): CMH 4 (2.2%): trauma	85 (47%): IVH 48 (27%): infection 13 (7.2%): tumours 31 (17%) temporal CSF derivation 4 (2.2%): trauma	N/A	N/A	N/A	Chest/Abdomen	N/A	N/A
Leung et al	114 Patients 133 LTEVD	Pediatric/Adult	84 (73.7%): OH 16 (14%): CMH 8 (7%): trauma (Drainage > 7 days)	57 (50.0%): IVH/SAH 23 (20.2%): ICH 8 (7.0%): trauma 7 (6.1%): shunt infection 9 (7.9%): infection others 4 (3.5%): tumours 6 (5.3%): other	N/A	N/A	40–50 + 10 at exit site for LTEVD	Chest	Rickham	N/A
Januschek et al	5 Patients	Pediatric	OH	IVH	N/A	N/A	N/A	N/A	N/A	N/A
Emelifeonwu et al	1 Patients	Pediatric	OH	Tumor	1 (100%) After VPS and VAS	35	30	Abdomen	Ommaya	N/A
Khanna et al	100 Patients 106 LTEVD	Pediatric/Adult	72 (72%): OH 14 (14%): CMH 8 (8%): trauma	26 (26%): IVH, BGH 22 (22%): SAH 14 (14%): tumor 14 (14%): infections 8 (8%): trauma 6 (6%): AVM 10 (10%): other ICH	39 (37%): from STEVD 10 (9%): from VPS	N/A	50–60 for LTEVD	Chest/Abdomen	Rickham	N/A
He et al	2 STEVD converted into LTEVD	Adult	OH (Drainage > 7 days)	BGH	1 (100%): from STEVD	2	N/A	Abdomen	N/A	Valve
LTEVD vs STEVD										
Chen et al	43 total 17 LTEVD 26 STEVD	Adult	CMH	Infection	N/A	N/A	N/A	Abdomen	N/A	N/A
Saenz et al	134 total 67 LTEVD 67 STEVD	Pediatric	114 (85,1%): CMH 18 (13,4%): OH 2 (1.5%): trauma (Drainage > 3 days)	43 (32.1%): Transient Hydrocephalus 71 (53%): shunt infection 13 (9.7%): IVH 2 (1.5%): Trauma 5 (3.7%): SDH Tumor; Postoperative; Infection*	N/A	≥ 1	20–25 for LTEVD 5 for STEVD	Chest/Abdomen	N/A	Valve
Sharma et al	38 total 11 LTEVD N/A STEVD	Pediatric/Adult	CMH	Infection	From STEVD	N/A	N/A	N/A	Ommaya	N/A
Soto-Hernández et al	92 total 34 LTEVD 58 STEVD	Adult	CMH	Infection	N/A	N/A	N/A	N/A	N/A	N/A
George et al	323 total 16 LTEVD 307 STEVD	Pediatric/Adult	CMH	Infection	N/A	11.6 ± 9.5	N/A	Chest	N/A	Valve
Tahir et al	60 total 30 LTEVD 30 STEVD	Pediatric/Adult	32 (53.3%): OH 28 (46,7%): CMH (LTEVD vs STEVD was the surgeon`s choice)	24 (40.0%): Infection 24 (40.0%): ICH 8 (13.3%): Tumor 4 (6.7%): Trauma	N/A	N/A	50–60 for LTEVD 5 for STEVD	Chest/Abdomen	N/A	N/A
Luo et al	187 total 82 LTEVD 105 STEVD	N/A	129 (68.98%): OH 41 (21,93%): trauma 17 (9.09%): CMH	129 (68.98%): ICH 41 (21.93%): trauma 17 (9.09%): infection	N/A	N/A	30 for LTEVD	Abdomen	N/A	Valve

**Table 2:** Overview of the outcomes of the 15 included studies

**Author**	P**atients** N	**Infection N (rate)**	**Dislocation N (rate)**	**Blockage N (rate)**	**CSF leakage N (rate)**	**Revision surgeries N (rate)**	**Mortality N (rate)**	**Mean drainage (days)**	OR **Removal (rate)**	S**hunt implantation N (rate)**
LTEVD										
Waqas et al	2 Patients	0	0	0	0	0	0	9.5 ± 0.5	N/A	0
Nakano et al	1 Patients	0	0	0	0	0	0	30	1 (100%)	1 VPS (100%)
Collins et al	177 Patients 181 LTEVD	5 (4%)	4 (2.3%)	0	N/A	13 (10%)	N/A	11.2 (0–42)	13 (9.8%)	VPS N. N/A
Leung et al	114 Patients 133 LTEVD	7 (6.8%)	N/A	9 (6.8%)	6 (4.5%): CSF leakage	14 (12%)	0	20 (1–60)	133 (100%) Under general anaesthesia/ local anaesthesia at chest	N/A
Januschek et al	5 Patients	1 (20%)	N/A	N/A	1 (20%): CSF leakage	N/A	0	> 14	N/A	N/A
Emelifeonwu et al	1 Patients	1 (100%)	N/A	1 (100%)	N/A	1 (100%)	0	N/A	N/A	N/A
Khanna et al	100 Patients 106 LTEVD	4 (4%)	N/A	6 (6%)	N/A	6 (6%)	0	N/A	N/A	VPS 62 (58%) Out of 106 LTEVD
He et al	2 STEVD converted into LTEVD	0	0	0	N/A	0	0	60	N/A	VPS: 1 (100)
LTEVD vs STEVD										
Chen et al	43 total 17 LTEVD 26 STEVD	0 (0%): LTEVD 7 (26.9%): STEVD	N/A	N/A	CSF leakage 0: LTEVD 8 (30.8%): STEVD	5 (29.4%): LTEVD 16 (61.5%): STEVD	N/A	N/A	N/A	VPS 5 (29%): LTEVD VPS 7 (27%): STEVD
Saenz et al	134 total 67 LTEVD 67 STEVD	2 (3%): LTEVD 15 (22.4%): STEVD	1 (1.5%): LTEVD 2 (3%): STEVD	1 (1.5%): LTEVD 7 (10.4%): STEVD	CSF leakage 6 (9%): LTEVD 14 (20.9%): STEVD	2.2 ± 0.3 (3.2%): LTEVD 2.1 ± 1.5 (3.1%): STEVD	N/A	19.2 ± 7.7: total 21.7 ± 7.9: LTEVD 16.7 ± 6.7: STEVD	LTEVD in the OR	N/A
Sharma et al	38 total 11 LTEVD N/A STEVD	N/A	N/A	N/A	N/A	N/A	N/A	11 (9.25–13.75): LTEVD 4 (3.5–4.5): STEVD	N/A	VPS 11 (100%): LTEVD
Soto-Hernández et al	92 total 34 LTEVD 58 STEVD	10 (29%): LTEVD 33 (57%): STEVD	N/A	N/A	N/A	N/A	N/A	N/A	N/A	N/A
George et al	322 total 16 LTEVD 307 STEVD	2 (13.3%): LTEVD* 5 (1.6%): STEVD	N/A	N/A	N/A	N/A	0	15.6 ± 9.9 (4–44)	N/A	VPS 10 (62.5%) ETV 1 (6.66%)
Tahir et al	60 total 30 LTEVD 30 STEVD	4 (6.7%): total 3 (10%): LTEVD 1 (3.3%): STEVD	N/A	N/A	N/A	N/A	1 (3.3%): LTEVD 6 (2%): STEVD	9.3 ± 6.8: total 13.4 ± 7.2: LTEVD 5.3 ± 2.7: STEVD	N/A	VPS 13 (43.3%): LTEVD VPS 10 (33.3%): STEVD
Luo et al	187 total 82 LTEVD 105 STEVD	4 (6.15%): LTEVD 13 (12.38%): STEVD	7 (8.54%): LTEVD 2 (1.90%): STEVD	3 (3.66%): LTEVD 5 (4.76%): STEVD	CSF leakage 0 (0%): LTEVD 5 (4.76%): STEVD	N/A	N/A	23.00 (14.00–33.50): LTEVD 7.00 (5.00–10.00): STEVD	N/A	N/A

**Table 3:** Additional information of the 15 included studies: demographics, definition of infection and quality assessment

Authors	Year	Study Design	Patients N		Male N (%)	Age Mean (months)	Definition of Infection	Definition and indications for revision surgeries	Quality Assessment*
LTEVD									
Waqas et al	2016	Case report	2		2 (100%)	4.25 (6–96)	N/A	N/A	N/A
Nakano et al	2007	Case report	1		1 (100%)	6	N/A	N/A	N/A
Collins et al	2014	Retrospective	177 LTEVD 181		N/A	79.2 (0–186)	“A novel bacterial growth on CSF or catheter during LTEVD placement, resulting in a one of the following changes in treatment: antibiotic therapy; change of EVD. It doesn’t consider already existing infections”	“Extra procedures required for LTEVD. Insertions and removal in the OR for existing infection (n = 48) were omitted”	4/1/3
Leung et al	2007	Retrospective	114 133 LTEVD		61 (54%)	631.2 (4–1080)	“Positive CSF culture does not present at catheter insertion. Of the 30 cases, 25 were old and 5 new. Most infections were found after 5–21 days of drainage.”	“Indication for revision were: > 7 days of drainage; extensive IVH; Infected shunts; Persistent infections; Catheter blockage; Loculated hydrocephalus”	3/0/3
Januschek et al	2011	Case report	5		N/A	6.37 gestation (pre-term)	N/A Infections most likely had abdominal origin	“Presence of leakage, repaired by suture, is an indication for revision surgery”	N/A
Emelifeonwu et al	2016	Case report	1		1 (100%)	4	“Lymphocytic pleocytosis and growth of bacteria on CSF subsequent cultures”	N/A	N/A
Khanna et al	1995	Retrospective	100 106 LTEVD		56 (56%)	624 (36–960)	“A positive CSF culture. If CSF cell count and glucose level were normal, and the patient was afebrile, bacterial growth was considered a contaminant”	N/A	4/0/3
He et al	2024	Case report	2 STEVD converted into LTEVD		1 (100%)	32	N/A	N/A	N/A
LTEVD vs STEVD									
Chen et al	2022	Retrospective	43 total 17 LTEVD 26 STEVD		10 (58.8%): LTEVD 14 (53.8%): STEVD	464.52 ± 188.4: LTEVD 481.8 ± 182.04: STEVD	N/A	N/A	4/1/3
Saenz et al	2023	Quasi experimental (LTEVD prospective, STEVD retrospective)	134 total 67 LTEVD 67 STEVD		73 (54.4%): total 36 (53.7%): LTEVD 37 (55.2%): STEVD	78 ± 64.8 67.2 ± 62.4: LTEVD 90 ± 66: STEVD	“A positive CSF culture after EVD insertion. Already existing infections were excluded except for the following circumstances: Initial infection was resolved; Negative CSF samples were obtained; Development of infection symptoms and new CSF sample positive for a microorganism different from the one isolated initially”	“Indication for revision were mechanical complications: Blockage – referred to as absence of CSF drainage > 12 h with no subsequent permeabilization; Displacement – referred to as system moved from the original position > 1 cm on imaging; > 3 CSF leakages; EVD-related infection. STEVD was routinely replaced every 13–15 days.”	4/2/3
Sharma et al	2019	Retrospective	38 total 11 LTEVD N/A STEVD		N/A	N/A	N/A	N/A	3/0/3
Soto-Hernández et al	2002	Retrospective	92 total 34 LTEVD 58 STEVD		N/A	N/A	N/A	N/A	3/1/3
George et al	2019	Retrospective	323 total 16 LTEVD 307 STEVD		10 (67%): LTEVD	198 (8–600): LTEVD	N/A	N/A	3/0/3
Tahir et al	2016	Prospective, non RCT	60 total 30 LTEVD 30 STEVD		32 (53%): total 17 (57%): LTEVD 15 (50%): STEVD	403,2 ± 292,8: total 333,6 ± 271,2: LTEVD 471,6 ± 302,4: STEVD	“(1) Rise in CSF leukocyte count above 10,000 cells/µl and temperature ≥ 38 °C; (2) Evidence of growth of any micro‑organism on CSF; (3) Evidence of growth of a new micro‑organism on CSF at the time of EVD.”	N/A	4/0/3
Luo et al	2023	Prospective, non RCT	187 total 82 LTEVD 105 STEVD		N/A	N/A	“Simultaneous presence of (1) to (4). (1) Clinical manifestation of infection. (2) Blood leukocyte count > 10 × 109/L or neutrophil ratio > 80%. (3) Abnormal intracranial pressure and CSF examination. (4) Imaging signs of infection (5) Positive CSF and other pathogenic tests. Old infections were excluded.”	N/A	3/1/3

Qualitative assessment of the abovementioned studies was organized into preoperative, intraoperative, and postoperative paragraphs

Vs, versus; N/A, not available; OH, obstructive hydrocephalus; CMH, communicating hydrocephalus; IVH, intraventricular hemorrhage; ICH, intracerebral hemorrhage; BGH, basal ganglia hemorrhage; SAH, subarachnoid hemorrhage; SDH, subdural haematoma; AVMr, arteriovenous malformations – ruptured; VPS, ventriculoperitoneal shunt; VAS, ventriculoatrial shunt; CSF, cerebrospinal fluid

* Only new infections were considered. Of the LTEVD group, one was wound infection and the other CSF infection

### Preoperative parameters and Indication for LTEVD

Indications for LTEVD varied across studies, but almost always included the long-term treatment of intracranial infections in patients requiring CSF drainage. One retrospective study [16] and 1 case report [10] stated that an indication for LTEVD was the anticipated necessity to drain cerebrospinal fluid (CSF) longer than 7 days. One quasi-experimental study states that the indication was a drainage duration of more than 3 days [25], while 1 prospective non-RCT states that the choice of LTEVD versus STEVD for CSF drainage was left to the surgeon’s discretion [29]. The most common indication, observed in almost 100% of the included studies, was hydrocephalus, often post-hemorrhagic in adults and obstructive in pediatric populations. Communicating hydrocephalus associated with CNS infections was also frequently reported. Four studies explicitly described LTEVD as a secondary procedure following prior shunt surgery or persistent infection after STEVD [8, 10, 14, 20]. Sharma et al. reports a STEVD to LTEVD conversion for continuous long-term CSF drainage in patients with Acinetobacter meningitis [26].

### Intraoperative parameters

The mean reported catheter tunneling length for LTEVD was 41.3 ± 14.0 cm, based on data from six studies [8, 14, 16, 18, 25, 29]. The catheter externalization site varied: five studies reported exclusively abdominal externalization [4, 8, 10, 18, 20]; four described either abdominal or thoracic externalization [6, 14, 25, 29] and two studies exclusively used thoracic externalization [9, 16]. Reservoirs reported were either Rickham [14, 16] or Ommaya types [8, 26]. Some studies utilized valve-assisted systems [9, 10, 18, 25].

### Postoperative parameters

#### Catheter removal and conversion to permanent CSF diversion

When indicated, removal of LTEVD occurs in the operating theatre under general anaesthesia [6, 16, 20, 25]. In contrast, STEVD was likely commonly removed bedside, though precise bedside removal rates were typically not provided [25]. As a permanent CSF diversion technique, ventriculoperitoneal shunt (VPS) was used in most cases [10, 14, 20, 26, 29]. The conversion rate to VPS following LTEVD, however, varied substantially across studies ranging from 9.8% to 100% [4, 6, 9, 14, 16, 20, 25, 26, 29]. Comparative conversion rates between LTEVD and STEVD were reported explicitly by Chen et al., who described VPS conversion rates of 29% (5 of 17) following LTEVD versus 27% (7 of 26) following STEVD [4].

#### Cerebrospinal fluid (CSF) leakage

CSF leakage was frequently reported as a postoperative complication in studies evaluating outcomes following LTEVD and STEVD placement [4, 13, 16, 18]. Leakage rates varied significantly, ranging from 4.5%, as reported by Leung et al. [16], to as high as 29.4%, as documented by Chen et al. [4]. Such complications commonly necessitated catheter revision or additional surgical intervention, with reported revision surgery rates varying from 3 to 30% in patients undergoing LTEVD [4, 6, 8, 14, 16, 25].

Several studies specifically compared CSF leakage rates between LTEVD and STEVD procedures. Chen et al. [4] noted significantly higher CSF leakage rates in STEVD patients (26.9%, 7 of 26 patients) compared to no leakage events (0%, 0 of 17 patients) following LTEVD insertion. Similarly, Saenz et al. [25] reported higher leakage incidence in STEVD (20.9%) compared to LTEVD patients (9%) [25]. Luo et al. [18] also found a higher CSF leakage rate in STEVD patients (4.8%) compared with LTEVD (0%) [18]. Collectively, these comparative findings suggest a consistent trend toward lower CSF leakage rates following LTEVD compared to STEVD.

Vs, versus; N, number; N/A, not available; LTEVD, long tunneled exernal ventricular drainage; STEVD, short tunneled external ventricular drainage; OR, operating room; VPS, ventriculoperitoneal shunt; VAS, ventriculoatrial shunt; CSF, cerebrospinal fluid; ETV, endoscopic third ventriculostomy; CNS, central nervous system.

#### Catheter dislocation and blockage

Catheter dislocation and blockage were reported complications following both LTEVD and STEVD procedures, with rates varying across the included studies. For LTEVD, reported dislocation rates ranged from 1.5% to 8.5%, while rates following STEVD insertion were between 1.9% and 3% [18, 25]. Comparative data revealed no consistent trend regarding dislocation rates, with Saenz et al. [25] reporting lower dislocation rate for LTEVD compared to STEVD (1.5% vs. 3%), whereas Luo et al. found interestingly a higher dislocation incidence with LTEVD (8.5%) than STEVD (1.9%) [18]. In both comparative studies, catheter blockage rates tended to be lower with LTEVD, ranging from 1.5% to 6.8%, compared to higher rates reported for STEVD, ranging between 4.8% and 10.4% [18, 25].

The chi-square analysis of the combined cohorts comparing blockage and dislocation rates between patients undergoing LTEVD and STEVD insertion found no statistically significant differences for either complication. Blockage rates were lower in the LTEVD group (2.7%) compared to STEVD (7.0%), but this difference did not reach statistical significance (p = 0.075). Dislocation rates were slightly higher in LTEVD (5.4%) than in STEVD (2.3%), yet this difference was also not significant (p = 0.151). Overall, both blockage and dislocation rates showed no clear advantage of one technique over the other.

#### Postoperative catheter infection

Infection rates for LTEVD across individual studies ranged from as low as 0% to as high as 29% [4, 6, 9, 14, 16, 18, 25, 27, 29]. Comparative studies demonstrated substantial variability (I^2^ = 74.6%). Qifu et al. and Saenz et al. observed markedly lower infection rates with LTEVD compared to STEVD (0% vs. 26.9% [4] and 3% vs. 22.4% [25], respectively). Luo et al. also reported lower infection incidence for LTEVD (6.2%) compared to STEVD (12.4%)[18]. Soto-Hernández et al. documented relatively high infection rates for both techniques (LTEVD: 29%; STEVD: 57%) [27]. Interestingly, two studies reported higher infection rates following LTEVD insertion compared to STEVD: George et al. found infection rates of 13.3% versus 1.6% [9], while Tahir et al. reported 10% versus 3.3%, both advocating for STEVD insertion. This last study was also the only one reporting EVD-related deaths: 3.3% for LTEVD and 2% for the STEVD group [29].

**Fig. 2:**
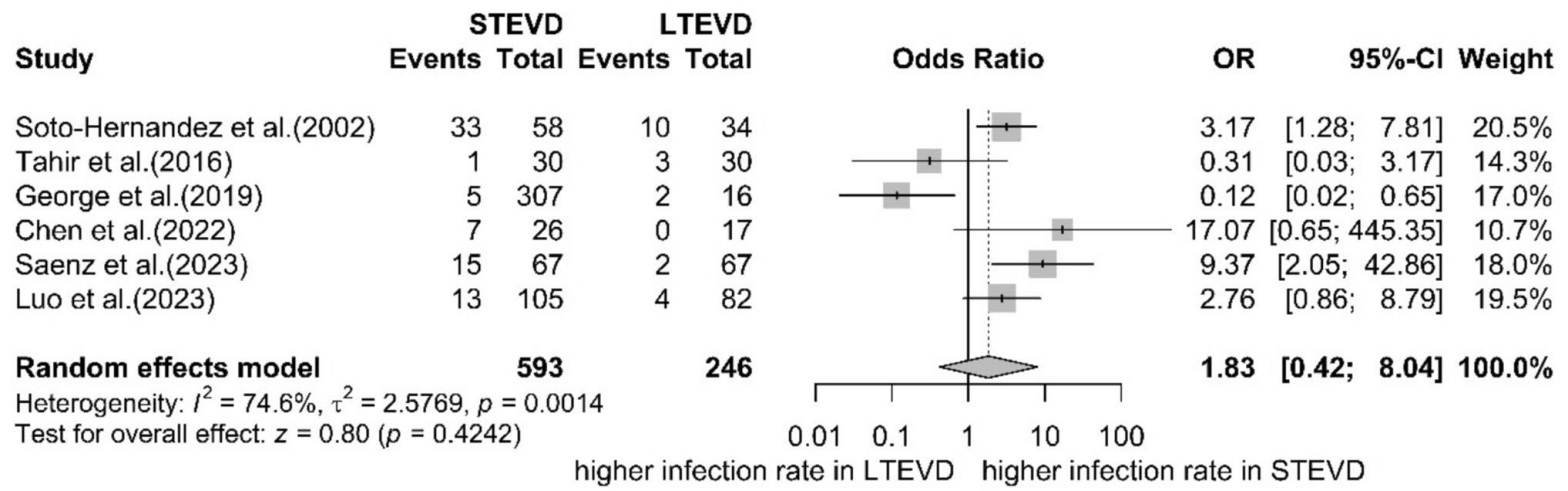
Forest plots detailing the meta-analysis of infection rates in the few studies comparing STEVD and LTEVD

**Fig. 3:**
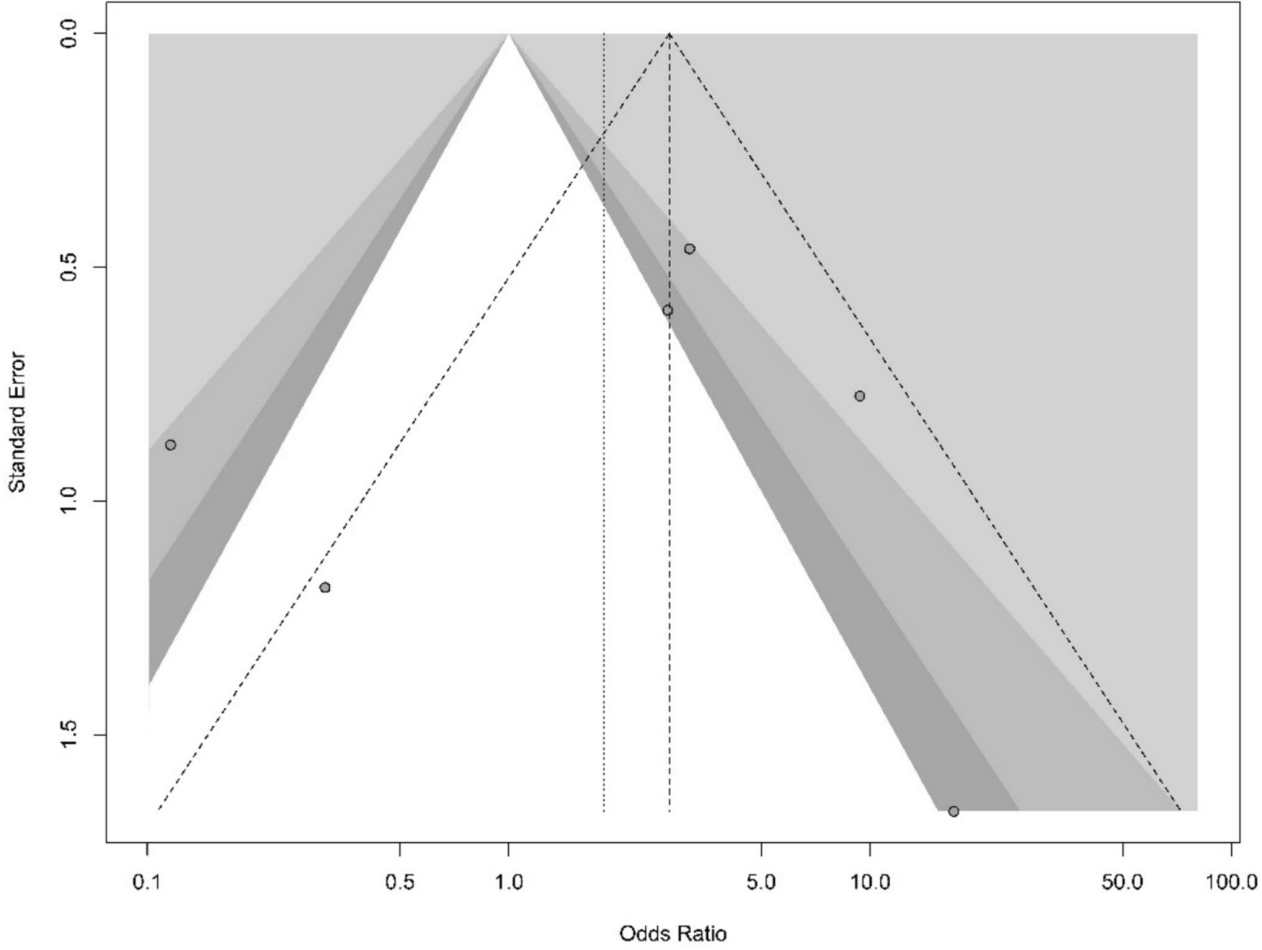
Funnel plot showing publication bias, only 6 Studies analyzed

A meta-analysis evaluating postoperative infection rates was feasible for six comparative studies (total n = 824 patients) [4, 9, 18, 25, 27, 29]. Due to considerable heterogeneity across studies, the pooled analysis revealed no statistically significant difference in infection rates between LTEVD and STEVD cohorts (Odds Ratio 1.83; 95%CI 0.84–3.99;p = 0.13) (Fig. 2 and 3).

## Discussion

Across the included studies, LTEVD was most commonly indicated for long-term CSF diversion in hydrocephalus and CNS infections, with variable surgical techniques regarding tunneling length, externalization site, and reservoir use/type. Postoperative outcomes demonstrated heterogeneous but generally comparable complication and conversion rates between LTEVD and STEVD, with a consistent trend toward lower CSF leakage and, in several studies, lower infection rates following LTEVD.

Catheter-related infection remains one of the most frequently reported complications associated with STEVD insertion [6, 17]. Multiple factors are known to influence infection risk, such as duration of drainage [19], CSF leakage [15], repeated catheter manipulations, and routine catheter changes [31]. Despite various strategies to mitigate these risks, significant variability in infection rates persists, partly reflecting the absence of standardized guidelines for EVD placement and postoperative care [5]. Reported infection rates following STEVD vary widely, ranging from 0% to as high as 32%, with even higher rates documented in developing regions [2, 3, 12, 19, 21, 29]. Nevertheless, shorter tunneling distances, even as brief as 5 cm, appear beneficial in reducing infection risks in resource-limited settings [21].

LTEVD theoretically reduces infection risks by increasing the physical separation between the catheter’s intracranial entry site and its external exit point [6, 14, 16, 25]. Several early studies evaluating LTEVD reported relatively low postoperative infection rates, ranging from approximately 4% to 6.8%. More recent comparative studies further support potential reductions in infection rates with LTEVD. Saenz et al. demonstrated a significant reduction in infection rates from 22.4% with STEVD to 3% with LTEVD among pediatric patients [25]. Similarly, Luo et al. and Chen et al. observed lower infection incidences in patients treated with LTEVD compared to STEVD [4, 18]. In contrast, two studies reported paradoxically higher infection rates associated with LTEVD. George et al. documented infection rates of 13.3% for LTEVD compared to 1.6% for STEVD, and Tahir et al. reported infection rates of 10% versus 3.3%, respectively [9, 29]. Tahir et al. suggested that LTEVD may delay rather than prevent infections, though this hypothesis was not supported by statistically significant findings.

To quantitatively assess the comparative infection risk between LTEVD and STEVD, we performed a meta-analysis of six comparative studies. Despite the observed trend toward lower infection rates in certain individual studies, pooled results did not demonstrate a statistically significant difference between LTEVD and STEVD cohorts (OR 1.83; 95% CI 0.84–3.99; p = 0.13). This lack of statistical significance is in part attributable to substantial heterogeneity and limited sample sizes across studies. Mixed patient populations, methodological variability, and inconsistent catheter management protocols further limited our ability to draw definitive conclusions.

LTEVD may provide particular benefits in scenarios requiring prolonged CSF drainage without increasing morbidity. Khanna et al. reported no infections over a mean drainage duration of 18 days, indicating that LTEVD could safely allow extended drainage durations [14]. In contrast, George et al. described increased infections in LTEVD patients after prolonged drainage of up to 44 days, highlighting potential risks associated with excessively prolonged catheter use [9]. As previously discussed, observational evidence consistently identifies duration of drainage and catheter manipulations as significant infection risk factors [1, 24]. By increasing tunneling length and thus reducing external colonization, LTEVD may theoretically allow prolonged drainage durations at lower infection risk. Nonetheless, variability in clinical practice and the absence of robust prospective data preclude definitive conclusions regarding optimal drainage duration when using LTEVD.

Beyond the aspect of infection alone, LTEVD insertion consistently demonstrated lower rates of CSF leakage compared to STEVD. CSF leakage significantly increases infection risks and often necessitates surgical revision, complicating clinical management, and prolonging hospitalization. Therefore, observed reductions in leakage rates with LTEVD represent a clinically meaningful benefit, especially for patients at high risk or requiring prolonged drainage periods, situations where minimizing CSF leakage is crucial. Similarly, LTEVD insertion was associated with consistently lower rates of catheter blockage compared with STEVD, ranging between 1.5% and 6.8% for LTEVD versus 4.8% to 10.4% for STEVD [18, 25]. Catheter dislocation rates were more variable across studies without clear superiority of either technique, although the dislocation rate was somewhat higher for LTEVD. Anecdotally, the authors offer an explanation for this due to the differing anchoring of LTEVD vs. STEVD: Namely, LTEVDs without an anchor proximally such as a Rickham reservoir (as used in some of the reported series) can proximally dislocate upon neck and torso movements, which is not the case with STEVD (which is directly anchored to the scalp and does not extend to the neck area, thus not being affected by neck and torso movements).

The observed reductions in blockage rates may be clinically relevant, as catheter occlusions frequently necessitate revision surgeries, potentially impacting patient morbidity and healthcare costs. LTEVD may thus be particularly advantageous in situations where catheter patency is imperative. A practical consideration associated with LTEVD involves the routine need for surgical removal under general anesthesia, especially when implanted reservoirs or valves are used [9, 18, 25]. Furthermore, our anecdotal experience suggest that the practical handling of significantly longer catheters without burr-hole reservoir occasionally used in LTEVD can be technically challenging during insertion, potentially complicating the procedure. One possible solution to mitigate this could involve using a Cushing-Cairns ventricular puncture cannula to initially perform the ventriculostomy, followed by separate insertion of the catheter. The potential clinical benefits of reduced infections, blockage rates, and CSF leakage may outweigh these additional surgical measures, underlining that a careful, individualized assessment of patient-specific factors and institutional resources is critical when considering LTEVD implementation.

Vs, versus; N/A, not available; J., Journal; EVD, external ventricular drainage; LTEVD, long-tunneled external ventricular drainage; STEVD, short tunneled external ventricle drainage; CSF, cerebrospinal fluid.

* Quality Assessment according to Newcastle–Ottawa Assessment Scale (NOS).

Our findings highlight substantial gaps and variability within existing literature. Prospective randomized controlled trials employing standardized protocols and clearly defined outcome measures are needed to clarify comparative effectiveness between LTEVD and STEVD. Such robust studies will be essential for developing evidence-based guidelines for EVD insertion techniques and catheter management strategies.

## Limitations

This present review and meta-analysis has several limitations that warrant acknowledgment. First, the quantity of evidence is limited, with only 15 studies and relatively small sample sizes. The lack of randomized evidence means our conclusions rely on observational data, which is subject to bias. Second, there was notable heterogeneity in patient populations, adjunct measures (antibiotic catheters, protocols), and outcome definitions across studies. We attempted to account for this via random-effects models, but heterogeneity remains, and our results should be interpreted with caution. Third, our primary focus was on infection outcomes; other potential benefits or drawbacks of long tunnels (like patient comfort, ease of nursing care, cost implications) were not well reported in the included studies and thus not analyzed here. Neither were potential confounders such as catheter antibiotic impregnation. Fourth, publication bias is possible: If small studies that showed no benefit of long EVD were less likely to be published, our review might overestimate any positive effect. We did not find strong evidence of publication bias, but the power was low. Another consideration is patient selection and indication bias. In some hospitals, LTEVDs might be reserved for patients deemed at very high risk of infection (e.g. those with prior ventriculitis or those expected to need a long duration of CSF diversion), whereas STEVDs might be used for more routine cases. If so, comparisons could be biased against long tunnels. Alternatively, some neurosurgeons might preferentially use LTEVDs in elective, controlled situation and use STEVDs in emergency bedside scenarios), which could bias results in favor of long tunnels because elective cases inherently might have lower infection risk. Unfortunately, the retrospective nature of most data makes it hard to eliminate such biases. Finally, the low overall quality of evidence means there is uncertainty in the effect estimates. For example, the confidence interval for infection reduction includes a meaningful benefit but also the possibility of little to no effect. Therefore, our conclusions should be seen as tentative.

Despite these limitations, this is the first comprehensive summary of LTEVD evidence. Our work consolidates the existing literature and provides a balanced perspective for clinicians. We highlight that while long-tunneled EVDs have intuitive and demonstrated advantages (delaying infection, possibly reducing dislodgement), they are not a guaranteed solution to prevent EVD-related infections. The findings underscore the importance of a multifaceted approach to EVD care. Long tunneling can be an additional possible measure alongside other standard components such as strict sterile technique during insertion and use of antibiotic-coated catheters to cumulatively reduce infection risk.

## Conclusion

Outcomes associated with LTEVD are generally comparable to conventional STEVD in pediatric and adult neurosurgical patients. Although comparative studies demonstrate a trend toward lower CSF infection rates with LTEVD, this finding did not reach statistical significance. Still, potential benefits such as reduced early postoperative infections and lower catheter blockage rates must be interpreted cautiously due to the limited methodological quality, substantial heterogeneity, and potential biases in the available literature. Thus, while routine adoption of LTEVD as a superior technique is perhaps not supported by current evidence, it is a viable and safe technique in the neurosurgeon’s armamentarium. The decision to utilize LTEVD should remain individualized, guided by patient-specific factors, expected duration of CSF drainage, and institutional protocols. It may be used with equipoise in situations where long-term external ventricular drainage is required, such as in the management of shunt infections with obstructive hydrocephalus.

## Data Availability

No datasets were generated or analysed during the current study.

## Declarations

### Registration

Not registered in PROSPERO.
